# The First Swiss National Nutrition Survey in Children and Adolescents, menuCH-Kids: Study Design, Participants, and Data Quality

**DOI:** 10.3389/ijph.2026.1609314

**Published:** 2026-02-17

**Authors:** Julia Vincentini, Julien Riou, Tanja Häusermann, Joëlle Schwitzguebel, Sandrine Estoppey Younes, Loan Catalano, Christine Brombach, Aziz Chaouch, Angeline Chatelan, Julia Dratva, Franziska Isler, Pascal Müller, Serge Rezzi, Franziska Righini-Grunder, Sabine Rohrmann, Christoph Saner, Giacomo D. Simonetti, Katja Uhlmann, Federica Vanoni, Christine Anne Zuberbuehler, Aline Siegfried-Troxler, Suzanne Suggs, Klazine van der Horst, Murielle Bochud

**Affiliations:** 1 Department of Epidemiology and Health Systems, Unisanté, University Center for Primary Care and Public Health and University of Lausanne, Lausanne, Switzerland; 2 School of Health Professions, Bern University of Applied Sciences, Bern, Switzerland; 3 Institute of Food technology, Zurich University of Applied Sciences, Wädenswil, Switzerland; 4 Department of Nutrition and Dietetics, Geneva School of Health Sciences, HES-SO University of Applied Sciences and Arts Western Switzerland, Carouge-Geneva, Switzerland; 5 Institute of Public Health, Zurich University of Applied Sciences, Winterthur, Switzerland; 6 YouGov Schweiz, Zürich, Switzerland; 7 Adolescent Medicine and Paediatric Psychosomatics, Children’s Hospital of Eastern Switzerland, St. Gallen, Switzerland; 8 Swiss Nutrition and Health Foundation, Lausanne, Switzerland; 9 Children’s Hospital of Central Switzerland, Lucerne, Switzerland; 10 Epidemiology, Biostatistics and Prevention Institute (EBPI), University of Zurich, Zurich, Switzerland; 11 Department of Pediatrics, Inselspital - Bern University Hospital and University of Bern, Bern, Switzerland; 12 Paediatric Institute of Southern Switzerland, Regional Hospital of Bellinzona - EOC and University of Lugano, Bellinzona, Switzerland; 13 Division of Food and Nutrition, Federal Food Safety and Veterinary Office, Bern, Switzerland; 14 Faculty of Communication, Culture and Society, University of Lugano, Lugano, Switzerland

**Keywords:** 24-hours dietary recalls, children and adolescents, nutrition survey, participation bias, Switzerland

## Abstract

**Objectives:**

menuCH-Kids was launched to generate the first Swiss nationwide children’s dietary data, assess food contaminant exposure, and inform nutrition policies. This paper describes the methods, data quality, and participants characteristics.

**Methods:**

In 2023–2024, a cross-sectional population-based survey in six Swiss centres collected dietary data via two non-consecutive 24-hour recalls/records and a Food Propensity Questionnaire; lifestyle, health, eating behaviours and sociodemographic information via online questionnaires; anthropometrics, urine, and voluntary blood samples by trained professionals with standardized procedures in 6–17-year-olds. Area-based socioeconomic position (Swiss-SEP) was linked to home addresses. Statistical weights corrected for unequal selection probabilities and non-response. Factors associated with participation were explored using logistic regressions.

**Results:**

1,852 participants attended the visit (participation rate = 11.9%). Data quality was high (<6% missing values, 15.1% dietary under-reporters, and 98% of biosamples processed on time). Non-participants were older, male, non-Swiss, from lower socioeconomic neighbourhoods, and smaller household. Adding socioeconomic position improved participation prediction models.

**Conclusion:**

menuCH-Kids provides high-quality dietary and health data on Swiss youth. Low participation highlights the need for a weighting strategy including socioeconomic position to compensate biases.

## Introduction

Diet is a major determinant of human health, with seven of the ten main causes of death and lost quality of life worldwide linked directly or indirectly to diet [1, 2]. Nutrition affects cardiometabolic functions, body composition and mental health of children and adolescents [3–8]. Childhood dietary habits often persist [9], and childhood overweight increases the risk of obesity later in life and thus type2 diabetes, cardiovascular disease and cancer [10]. European youth diet are often insufficiently healthy, with low vegetables intake and excessive intakes of meat, fat and sugar [3, 11]. Children overweight is 10%–35% in most European countries [12, 13], and 15% in Switzerland [14]. Children are also vulnerable to food contaminants [15], and risk assessments should be grounded in national dietary habits and behaviours.

To promote healthy lifestyles starting early and reduce nutrition-related diseases later, in-depth knowledge of the country’s current youth situation is essential. Given the observed differences in dietary habits across Europe [16], country-specific data are essential to inform nutrition policies adapted to the local context. Despite its small size, Switzerland is diverse in culture, languages spoken, and dietary traditions. The 2015 adult national nutrition survey menuCH (2,000 adults aged 18–75), revealed regional dietary differences across Switzerland’s linguistic regions [17]. To date, no nationally representative data on children’s and adolescents’ diets and behaviours were available in Switzerland.

menuCH-Kids, the first Swiss national nutrition survey of children and adolescents, fills this gap. It was commissioned by the Federal Office of Food Safety and Veterinary Affairs (FSVO) and conducted by 15 institutions. This multicentric, cross-sectional survey collected representative, high-quality data on diet, nutritional status, behaviours, lifestyle, anthropometrics, and blood and urine from 6 to 17-year-olds. Its design adheres to Swiss-specific and European recommendations [18, 19], ensuring standardized, comparable data, and covers all linguistic regions.

This paper primarily describes the design, methodology, and available data of the menuCH-Kids survey, and documents participants’ characteristics and participation bias. Data reliability is illustrated by selected descriptive indicators.

## Methods

### Study Design

#### Setting

The survey was commissioned by FSVO and conducted in six centres, covering Switzerland’s three main linguistic regions (i.e., German, French, Italian): Bern University Hospital (Bern - BE), Children’s Hospital of Central Switzerland (Lucerne - LU), Children’s Hospital of Eastern Switzerland (St Gallen - SG), Regional Hospital of Bellinzona (Ticino - TI), Unisante - University Centre for Primary Care and Public Health (Vaud - VD), University of Zurich (Zurich - ZH). Participants were recruited by the survey institute YouGov® Schweiz. Four institutions handled the biosamples (Liquid Biobank Bern (LBB) for storage, Swiss Nutrition and Health Foundation (SNHf) for vitamin analyses, Federal Institute of Metrology (METAS) for trace elements analyses, Centre Hospitalier Universitaire Vaudois (CHUV) for other analyses). Additional institutions provided counselling and quality control (see Supplementary Material 1 for organisation chart). Participant recruitment and data collection were conducted in German, French, and Italian.

#### Timeline and Ethical Approval

Initiated in 2020, the project had a pilot phase in the spring 2023, after receiving approval from research ethics committees of all relevant cantons (lead committee in Lausanne, protocol n∘ 2022-01602, approved on 09.02.23). The main data collection phase lasted from September 2023 to September 2024. Informed consent was given by both children and parents for children under 13 years old, whereas adolescents from 14 years old could come and sign alone.

#### Pilot Survey

A pilot survey with 186 participants (Feb-Jun 2023) tested all procedures planned for the main study. A 2017 feasibility study already assessed the acceptability of biosamples and data collection in children [20]. The pilot evaluated protocol adequacy across all study centres, suitability of the settings and data and biosamples quality. It confirmed the feasibility of standard operating procedures, participant recruitment, and a well-functioning governance structure. We fine-tuned the communication strategy, activity timing, and study documents based on fieldworkers’ feedback, but no methodological adjustments were needed, except for the discontinuation of Biozoom® β-carotene skin scan use due to poor accuracy and reliability.

### Participants and Recruitment

#### Sampling

Invited people were randomly selected from the Swiss cantonal population registries via the Federal Statistical Office (FSO) sampling frame (SRPH) [21]. First, municipalities (N = 950) within a 30-km drive to one of the six study centres were selected. Then, a stratified sample by centre and age category (6–9, 10–13, 14–17 years old) created 18 strata. Four waves were drawn over a year, allowing for continuous recruitment using the most current version of the registry while capturing seasonal variations. Assuming a 10%–15% participation rate, as in menuCH adult and previous Swiss surveys, 12,024 addresses were selected, with an additional 6,036 in reserve, to reach the 1,800 participants target.

Centre-specific participant targets roughly reflected Switzerland’s population density, while ensuring enough participants from each linguistic region. This implied oversampling the Italian-speaking canton of Ticino and reducing sampling of the Zurich region, which had the largest eligible population. Complete participation targets were: 390 in Vaud and Bern, 300 in Zurich, 270 in St Gallen, 240 in Lucerne, and 210 in Ticino. Although equal age group distribution was planned, due to some communication problem, the FSO-provided sample included 40% aged 6–9, 26% aged 10–13, and 34% aged 14–17. Once identified, this imbalance was maintained across seasons, for consistency, and as 10–13-year-olds showed higher participation (Table 1), and 6–9-year-olds lower blood draw acceptance (Table 2).

**TABLE 1:** Characteristics of the sample based on participation status and population proportions (in eligible children^c^) (menuCH-Kids survey, Switzerland, 2023–2024).

Characteristics	Participants N = 1,852^a^	Non-participants N = 14,371^a^	p-value^b^	Invited sample N = 16,223^a^	Population^c^ proportions
Age category	​	​	<0.001	​	​
6–9	748 (40%)	5,701 (40%)	​	6,449 (40%)	36%
10–13	560 (30%)	3,702 (26%)	​	4,262 (26%)	35%
14–17	544 (29%)	4,968 (35%)	​	5,512 (34%)	30%
Study centre	​	​	0.17	​	​
BE	394 (21%)	2,859 (20%)	​	3,253 (20%)	18%
LU	242 (13%)	1,767 (12%)	​	2,009 (12%)	14%
SG	277 (15%)	1,971 (14%)	​	2,248 (14%)	12%
TI	214 (12%)	1,749 (12%)	​	1,963 (12%)	5%
VD	408 (22%)	3,395 (24%)	​	3,803 (23%)	16%
ZH	317 (17%)	2,630 (18%)	​	2,947 (18%)	35%
Biological sex	​	​	0.029	​	​
Female	942 (51%)	6,923 (48%)	​	7,865 (48%)	49%
Male	910 (49%)	7,448 (52%)	​	8,358 (52%)	51%
Nationality	​	​	<0.001	​	​
Swiss	1,600 (86%)	10,592 (74%)	​	12,192 (75%)	76%
Other	252 (14%)	3,779 (26%)	​	4,031 (25%)	24%
Socioeconomic level quintiles	​	​	<0.001	​	​
1	283 (15%)	3,314 (23%)	​	3,597 (22%)	20%
2	351 (19%)	2,945 (20%)	​	3,296 (20%)	20%
3	404 (22%)	2,841 (20%)	​	3,245 (20%)	20%
4	404 (22%)	2,800 (19%)	​	3,204 (20%)	20%
5	410 (22%)	2,471 (17%)	​	2,881 (18%)	20%
Household size	​	​	<0.001	​	​
2	41 (2%)	656 (5%)	​	697 (4%)	4%
3	260 (14%)	2,518 (18%)	​	2,778 (17%)	16%
4	918 (50%)	6,688 (47%)	​	7,606 (47%)	46%
5	471 (25%)	3,163 (22%)	​	3,634 (22%)	23%
6+	162 (9%)	1,346 (9%)	​	1,508 (9%)	10%
Residential area	​	​	<0.001	​	​
Urban	1,143 (62%)	9,512 (66%)	​	10,655 (66%)	-
Suburban	442 (24%)	3,219 (22%)	​	3,661 (23%)	-
Rural	267 (14%)	1,640 (11%)	​	1,907 (12%)	-

^a^ n (%).

^b^ Pearson’s Chi-squared test.

^c^ Proportions in our eligible population, from 6 to 17 years old and around the six study centres.

**TABLE 2:** Characteristics of participants with and without blood draw (menuCH-Kids survey, Switzerland, 2023–2024).

Characteristics	Blood draw N = 848^a^	No blood draw N = 1,004^a^	p-value^b^
Age category	​	​	<0.001
6–9	266 (31%)	482 (48%)	​
10–13	287 (34%)	273 (27%)	​
14–17	295 (35%)	249 (25%)	​
Study centre	​	​	>0.99
BE	186 (22%)	208 (21%)	​
LU	111 (13%)	131 (13%)	​
SG	125 (15%)	152 (15%)	​
TI	98 (12%)	116 (12%)	​
VD	186 (22%)	222 (22%)	​
ZH	142 (17%)	175 (17%)	​
Biological sex	​	​	0.49
Female	424 (50%)	518 (52%)	​
Male	424 (50%)	486 (48%)	​
Nationality	​	​	0.85
Swiss	734 (87%)	866 (86%)	​
Other	114 (13%)	138 (14%)	​
Socioeconomic level quintiles	​	​	0.19
1	137 (16%)	146 (15%)	​
2	144 (17%)	207 (21%)	​
3	183 (22%)	221 (22%)	​
4	199 (23%)	205 (20%)	​
5	185 (22%)	225 (22%)	​
Household size	​	​	0.049
2	17 (2%)	24 (2%)	​
3	104 (12%)	156 (16%)	​
4	412 (49%)	506 (50%)	​
5	228 (27%)	243 (24%)	​
6+	87 (10%)	75 (8%)	​
Residential area	​	​	0.84
Urban	525 (62%)	618 (62%)	​
Suburban	205 (24%)	237 (24%)	​
Rural	118 (14%)	149 (15%)	​

^a^ n (%).

^b^ Pearson’s Chi-squared test.

#### Recruitment and Participation Steps

The following steps were used to recruit participants, collect data, and provide compensation:

- An invitation letter, including a child-friendly flyer explaining the survey and a postcard addressed specifically to the child, followed by a reminder letter 2 weeks later when needed. For households without phone number provided by FSO (29.5%), a prepaid postcard was included to collect contact details.
- A recruitment call by a trained recruiter to assess eligibility (health, age, language, residency in Switzerland, see Supplementary Material 2) and schedule a visit for consenting participants, who then received study material and online questionnaire access.
- An on-site visit 2–10 weeks after the recruitment call included a 24-h dietary recall/record (visit 24HDR) with a registered dietitian, physical measurements and biosamples collection by a registered paediatric nurse, including voluntary blood draw. Children received a 20 CHF-voucher (book or cinema) as a thank-you for their participation.
- A second 24-h dietary recall/record on the phone (phone 24HDR) conducted with the dietitian, ∼2–4 weeks after the on-site visit.
- A mailing with vouchers (60.- CHF in holiday checks), personal results (selected physical measurements and blood results), and a booklet with dietary advice and recipes.

### Data Collection Procedures

#### Data Management

Personal data, contact information and recruitment data, were managed by YouGov® on their secured web-based platform. Coded online questionnaire responses were stored there, separately from personal data, and with restricted access. All non-identifying sociodemographic information from FSO, along with visit related data (e.g., biosamples metadata, physical measurements, results of blood analysis) were entered into REDCap® in coded form, whereas the two 24HDRs were recorded using GloboDiet®. Socioeconomic position of respondents and non-respondents was estimated using the Swiss Neighbourhood Index of Socioeconomic Position (Swiss-SEP) [22, 23], which assigns a score to each residential building (see Supplementary Material 3).

#### Online Questionnaire

Recruited households completed a pre-tested self-administered ∼30-item online questionnaire (see Supplementary Material 4). For all children and adolescents, parents answered questions on the child’s early life, diet and health, sociodemographic, and household characteristics. Adolescents (14–17 years) answered independently questions on eating habits, food allergies, a short Food Propensity Questionnaire (18 foods, 12 beverages), dietary supplements, and lifestyle (physical activity, screen time, sleep, alcohol, smoking, pocket money). For children aged 6–13 years, these questions were included in the parent questionnaire (excluding alcohol, smoking, and pocket money), with parents encouraged to complete them jointly with their child. Answers accuracy was not checked, but completion was verified during the visit.

#### Food Consumption Data and Misreporting

Following the 2014 European Food Safety Authority (EFSA) guidelines [19], food consumption was recorded in a highly standardized way using two non-consecutive 24HDR with the multiple–pass software GloboDiet® (trilingual version adapted for Swiss foods, version 1.2023.01.06 based on version 0.2016.4.10), balancing the need to capture day-to-day dietary variability with minimizing participant burden. The first recall was conducted during the on-site visit and the second one on the phone (∼2.5 weeks later), by a trained dietitian. The 24-hour period spanned from waking-up the day before to waking-up on the interview day.

Children (6–13-year-olds) and their parents completed a food diary on the recorded day (24h record), to support the interview process. Adolescents (14–17-year-olds) completed 24h-recalls (without notes) and could be accompanied or not by a parent. Portion size quantification was supported by a picture book (129 series of four to six portion-size pictures or bread shapes) [24] and a standard set of ∼60 real dishes displayed in all centres, as in menuCH adult [17] (see updates in Supplementary Material 5). A balanced distribution across weekdays and seasons was applied to capture dietary variability.

Because GloboDiet® contains only limited food composition information, all reported foods and recipes were matched with the closest generic foods or recipes of the Swiss Food Composition Database, v6.5.3, using the FoodCase matching wizard [25, 26], to estimate energy and nutrient intake. Foods were also classified according to the new 2024 Swiss Food Pyramid subgroups [27] (see Supplementary Material 5).

Plausibility of 24HDR was assessed by calculating under- and over-reporters’ prevalence, applying Goldberg method adapted by Black [28, 29]. Energy intake (EI) was taken from the 24HDRs and Basal Metabolic Rate (BMR) computed using Schofield’s equations by age and sex [30]. Physical activity level (PAL) was estimated from questionnaire and classified as low, medium and high PAL, corresponding to age-specific values, as recommended by EFSA [31], and with medium assigned to missing and unplausible (e.g., “0 day”) PA [32] (see details and sensitivity analyses in Supplementary Material 6).

#### Physical Measurements

Most measurements were performed at the study centre by a trained dietitian or nurse, following Measurement Toolkit Guidelines from Cambridge University [33] and the World Health Organization (WHO) guidelines [34], the European Society of Hypertension practice guidelines [35], or device manuals.

- Weight and height were measured to the nearest 0.1 kg/cm on calibrated scales with stadiometers (SECA 704), with light clothing, empty pockets, and no shoes, ensuring a Frankfort plane for height. Weight was adjusted by subtracting clothing weight (depending on age and type of clothes, see Supplementary Material 7).
- Waist and hip circumferences were measured with a non-stretchable tape to the nearest 0.1 cm, at the mid-point between the lowest rib and the iliac crest on bare skin, and the largest part of the buttocks on pants. Mean waist and hip circumferences were calculated based on three consecutive measures, removing the tapes indicating anatomical landmarks between measurements.
- Bioimpedance measurements were performed in four centres owning a device clinically validated on children (TANITA MC-780 in Bern and Vaud; InBody 770 in Zurich and St. Gallen).
- Blood pressure was measured three times in a sitting position with the Omron 1320, on the non-dominant arm, with the appropriate cuff size after a five-minute rest. The mean of the two last measurements was used for analysis. If only two measurements were taken, the mean was used unless systolic values differed >20 mmHg or diastolic >10 mmHg, in which case the second reading was used.
- Skin type was defined using the Fitzpatrick scale type bar tool [36] on the inner side of the upper arm.
- Puberty stage was self-assessed at home using the illustrated 5-stages Tanner scale for breast and pubic hairs in girls, and testicle development and pubic hairs in boys, plus an additional question about menstruations (girls) and voice change (boys).
- Additional open questions about current medication use, dietary supplements, disease, fish consumption in the last 7 days, fasting state, and time spent outside were asked by the nurse.

#### Biosamples Collection and Management

All centres followed a standardised protocol for biosample collection and management. Biobanking processes were certified by the Swiss Biobanking Platform Vita label, and metadata were collected for traceability and quality control. Samples were identified solely by barcode to ensure de-identification.

Spot urine was collected at home using a cup sent by post with hygiene instructions. First morning urine and storage in the refrigerator were recommended. At the centre, ∼17 mL of urine was aliquoted into nine tubes and frozen at −80 °C, with temporary −20 °C storage if needed (e.g., during weekend).

Voluntary blood sampling was performed by a paediatric nurse, with 21.8 mL of venous blood collected in six tubes (2 serum, 3 EDTA and 1 Lithium-Heparin), for direct analysis of blood formula and glycated haemoglobin or for aliquoting and −80 °C freezing for further batch analyses and biobanking (see Supplementary Material 8 for details on preanalytical conditions, biobanking and analyses done). Fasting was recommended for appointments up to 9a.m.; 48% of participants were fasting. Due to budgetary constraints, a sample size of ∼800 people was set for voluntary blood collection.

#### Quality Control Procedures

Standard operating procedures were implemented across all centres to ensure data quality and interoperability. Five trainings were conducted with all staff (two before the pilot, one before the main phase, two refreshers). Performance and adherence were monitored through regular centres visits, both announced and unannounced, by senior staff and internal experts (two during the pilot, six during the main phase). Weekly to bi-monthly data cleaning, based on predefined criteria, enabled ongoing monitoring of field progress and detection and correction of mistakes and inconsistencies.

### Weighting and Statistical Analyses

#### Weighting Strategy

Three sets of sampling weights were constructed to align the participant sample with the Swiss population: one for the on-site visit sample (n = 1,852), one for the blood sample subgroup (n = 848) and one for all questionnaires, including those without a visit (n = 1,935). All sets were corrected for non-response and calibrated to population margins using sampling strata (study centre and age group), sex, household size, nationality, Swiss-SEP quintiles, and season (defined as 3-month periods from September 2023). On-site visit weights were further adjusted to reflect weekday distribution of 24HDRs (two 24HDRs during weekdays [Mon-Thurs], two during weekends [Fri-Sun], one of each). For single recalls, the day category was used.

All margins except Swiss-SEP, seasons, and weekdays were obtained by averaging the FSO sampling frames of the four invitation waves. Swiss-SEP margins used FSO counts of 6–17-year-olds per building, linked via EGID to the Swiss-SEP register (version3 [37]). Seasonal margins split the sampling frame into four equal parts; weekday distribution followed a 16/49 (week), 9/49 (weekend), and 24/49 (mixed) ratio. Extreme weights, defined as >5 × the median plus interquartile range, were trimmed to this limit. Variance estimation used a rescaling bootstrap (1,000 replicates per weight set) (See Supplementary Material 9).

To use the provided weights in future analyses, all participants need to be included, even those with missing data in certain variables, as excluding participants based on missingness would require computing specific weights each time. Thus, we suggest using multiple imputation by chained equations (MICE) [38], for its flexibility with variable types and ability to model complex interdependencies. A possible choice for the imputation model includes 56 variables capturing relevant socio-demographic and health information (See Supplementary Material 9). Of course, depending on the research question, other variables might be appropriate. Ten datasets can be generated, and final estimates pooled using Rubin’s rules to account for imputation and sampling uncertainty.

#### Statistical Analyses

For the participation analyses and data quality indicators, unweighted results have been used. Chi-square tests compared characteristics between participants and non-participants, and between participants with and without blood samples. Multiple logistic regression identified factors associated with participation. Model M1 included age group (ref: 6–9 years), sex (ref: female), study centre (ref: Vaud), nationality (ref: Swiss), household size (ref: 4), and residential area (ref: urban). Model M2 added Swiss-SEP quintile (ref: 5th). Model comparison used the likelihood ratio test (LRT) and Nagelkerke R^2^. Analyses were conducted in R version 4.2.2 (2023).

## Results

### Participation Rate

**FIGURE 1:**
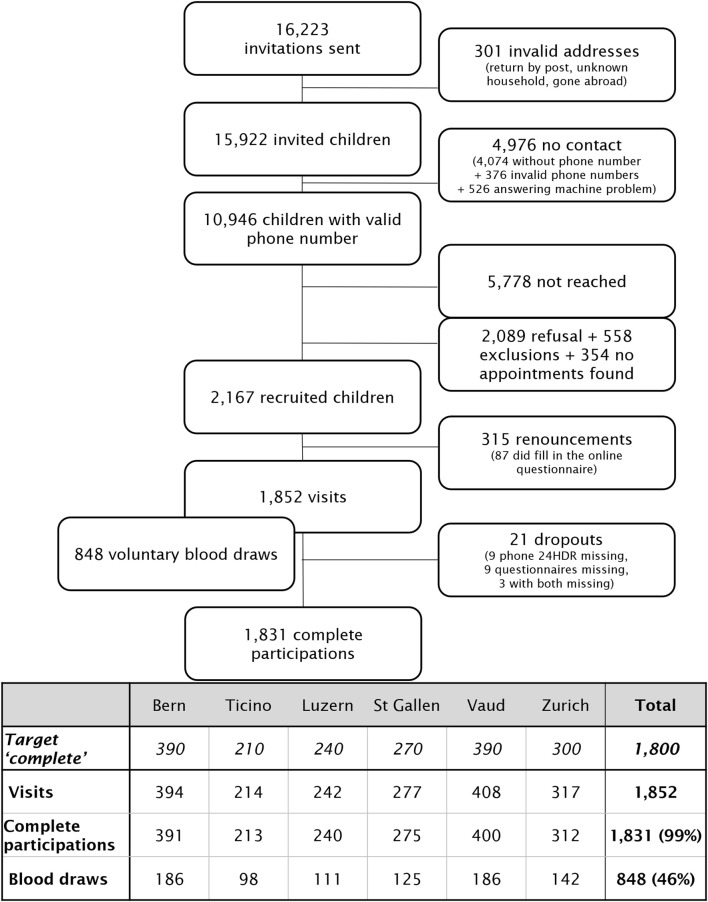
Recruitment flowchart (menuCH-Kids survey, Switzerland, 2023–2024).

During the main phase, 16,223 invitation letters were sent (Figure 1), with 15,364 eligible households after suppression of invalid addresses (N = 301) and households meeting exclusion criteria (N = 558). About two thirds of invited people were not reached by phone due to missing/unusable numbers (N = 4,976, 31.2%) or no answer (N = 5,778, 36.3%). Only 583 of the 4,704 households without available phone responded via prepaid contact postcards (12.4%), yielding 328 recruits. Among those reached by phone, refusals were mainly due to lack of interest (43%), lack of time (28%), and unsuitable time/date for the on-site visit (8%).

There are 1,852 participants (= with visit data collected), reaching the target of 1,800. Almost all participants (N = 1,831, 99%) completed all steps (online questionnaire, on-site visit, phone 24HDR), resulting in a participation rate of 11.9% (complete participations/eligible households), in the range of the expected 10%–15%. Additionally, 848 voluntary blood samples were collected (46% of the participants).

### Participant Characteristics

Socio-demographic characteristics significantly differed between participants (N = 1,852) and non-participants (N = 14,371) (Table 1). Participants were younger, of Swiss nationality, of higher socio-economic position, from a medium household size (4-5 people) and a rural environment.

Population proportions were derived from the FSO sampling frame (SRPH) from which invited households were selected (Table 1). Due to stratified sampling, age and study centre distributions do not reflect population proportions. Participant distributions of Swiss-SEP quintiles and nationality, and to a lesser extent, sex and household size, differ from the general population. No population data are available for residential area, as only invited addresses were linked to the FSO geographical area registry [39].

Blood draw acceptance depended on age, with a higher acceptance from older participants (54% vs. 36% in 6-9-year-olds), and slightly from larger households (Table 2). Study centre, sex, nationality, socioeconomic position and residential area did not affect blood sampling.

### Factors Associated With Participation

**FIGURE 2:**
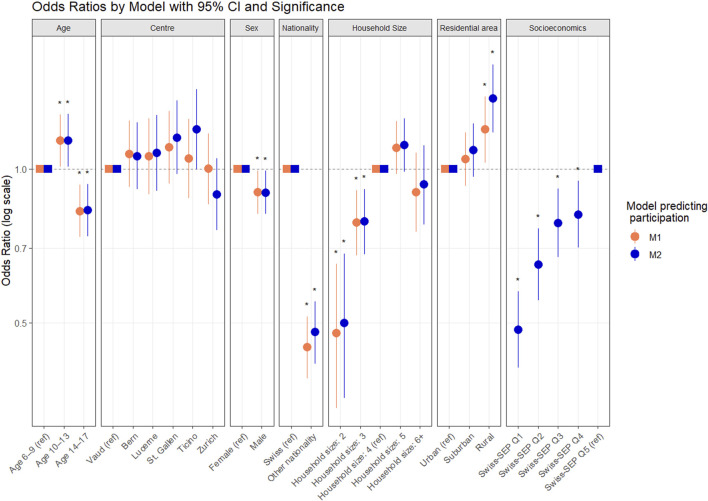
Influence of Socioeconomic position on participation: Comparison of logistic regression models. (menuCH-Kids survey, Switzerland, 2023–2024). M1: basic model predicting participation including age, centre, sex, nationality, household size and residential area; M2: model adding Swiss-SEP. Odds ratios with 95% confidence interval. * for significance.

Figure 2 presents factors associated with participation from multiple logistic regression models. In model M1, compared to children aged 6–9, those aged 10–13 had higher odds of participation, while those aged 14–17 had lower odds. Being male, non-Swiss, or from a smaller household was associated with lower participation (see details in Supplementary Material 10).

After adding Swiss-SEP (Model M2), associations remained largely unchanged, except for rural area becoming more strongly associated with higher participation. Lower Swiss-SEP was linked to lower participation (Figure 2). Including Swiss-SEP significantly improved model fit (LRT, *p* < 0.001), increased explanatory power (Nagelkerke R^2^ from 0.29 to 0.39), and augmented performance (AIC from 11,309.83 to 11,240.13), confirming the added predictive value of socioeconomic factors in the weighting strategy.

### Data Quality Indicators

#### Lifestyle Data Reliability

A few key unweighted lifestyle indicators were assessed to check data reliability. Body Mass Index (BMI) in the normal range (>P10 - <P90 using WHO percentiles) is 76%, with 13% overweight or obese. Among 14–17-year-olds, 11% reported smoking (including e-cigarette) and 36% drinking alcohol occasionally.

#### Quality of Dietary Recalls

Dietary data completeness was high, with 1,840/1,852 participants with two 24HDRs (12 phone 24HDR missing (0.6%)). Weekday distribution was balanced, though Fridays and Saturdays were slightly underrepresented (Table 3). While scheduling prioritised feasibility over weekday, 46% of participants completed one recall on a weekend and one on a weekday, 9% had both on weekends, and 11.9% had both recalls on the same day of the week. All seasons were well represented (Table 3). The average interval between recalls was 19 days (∼2.5 weeks). On average, 22 foods were reported during the visit 24HDR and 19 during phone 24HDR, with a significant decrease in mean energy intake (1,990 and 1,877 kcal/day, respectively).

**TABLE 3:** Description of the dietary data quality (menuCH-Kids survey, Switzerland, 2023–2024).

Dietary recall characteristics	Visit	Phone	Combined
Number of 24HDR	1,852	1,840	3,692
Weekdays distribution^a^			
Monday	334 (18.0%)	348 (18.9%)	682 (18.4%)
Tuesday	293 (15.8%)	316 (17.2%)	609 (16.4%)
Wednesday	293 (15.8%)	332 (18%)	625 (16.9%)
Thursday	279 (15.0%)	309 (16.8%)	588 (15.9%)
Friday	226 (12.2%)	128 (7%)	354 (15.1%)
Saturday	-	184 (10%)	184 (5%)
Sunday	427 (23.1%)	223 (12.1%)	650 (17.6%)
Seasons distribution^a^			
Spring (March - May)	495 (26.7%)	487 (26.5%)	982 (26.6%)
Summer (June- August)	486 (26.2%)	525 (28.5%)	1,011 (27.4%)
Autumn (September-November)	457 (24.7%)	441 (24.0%)	898 (24.3%)
Winter (December-February)	414 (22.4%)	387 (21.0%)	801 (21.7%)
Mean number of foods	22	19	20
Mean kcal/d	1,990	1,877	1,934

^a^ (n, %).

#### Energy Intake Plausibility and Under-reporting

Mean energy intake (average of two 24HDRs) increased with age and was higher in boys (Table 4). Mean EI:BMR ratio was 1.51, higher in younger children and in boys. Of the 1,852 participants, plausible reporters represent 84.3% (N = 1,561), under-reporters 15.1% (N = 279), more frequently older adolescents and girls, and over-reporters 0.6% (N = 12).

**TABLE 4:** Energy intake, Energy Intake/Basal metabolic rate ratio and under-reporting prevalence by age group and sex. (menuCH-Kids survey, Switzerland, 2023–2024).

Energy intake and reporting characteristics	Boys (N = 910)	Girls (N = 942)	Both sexes (N = 1,852)
Energy intake (kcal) - *mean (SD)*			
6–9-year-olds	1,878 (406)	1,650 (359)	1,764 (400)
10–13-year-olds	2,107 (491)	1,873 (453)	1,993 (487)
14–17-year-olds	2,439 (765)	1,827 (514)	2,107 (710)
Total	2,104 (595)	1,770 (450)	1,934 (552)
EI:BMR^a^ - *mean (SD)*			
6–9-year-olds	1.68 (0.35)	1.61 (0.35)	1.64 (0.35)
10–13-year-olds	1.52 (0.34)	1.49 (0.36)	1.50 (0.35)
14–17-year-olds	1.39 (0.44)	1.28 (0.38)	1.33 (0.41)
Total	1.55 (0.39)	1.47 (0.39)	1.51 (0.39)
Prevalence of under-reporters			
6–9-year-olds	1.9%	4.5%	3.2%
10–13-year-olds	13.9%	13.9%	13.9%
14–17-year-olds	28.1%	36.3%	32.5%
Total	12.9%	17.2%	15.1%
Prevalence of over-reporters			
6–9-year-olds	1.1%	1.1%	1.1%
10–13-year-olds	0.0%	0.0%	0.0%
14–17-year-olds	1.6%	0.0%	0.7%
Total	0.9%	0.4%	0.6%

^a^ Energy intake over basal metabolic rate.

#### Quality and Preanalytical Conditions of Biosamples

High traceability in sampling and processing enabled assessment of adherence to pre-analytical protocols and sample quality. For blood, the mean sampling-centrifugation time was 33 min, and sampling to −80 °C freezing, 58 min. Fifteen of 848 samples (2%) exceeded 3 hours before freezing, potentially affecting sensitive biomarkers [40], although storage on ice and in the dark may have mitigated degradation. In 78% of blood draws, all 26 aliquots were successfully completed. In 9%, not all blood collection tubes could be taken, resulting in incomplete sampling. In 13%, some aliquots are missing while collection was complete due to insufficient volume in some collection tubes. Missing data for blood parameters already analysed remain below 6%. Among the 1,852 participants, seven (0.4%) lack urine sample, 26 (1.4%) have incomplete aliquots, and 6 (0.3%) were processed late. Most participants (1,685, 91%) provided first-morning urine samples.

## Discussion

Switzerland’s first national nutrition survey among 6–17-year-olds, menuCH-Kids, offers an exceptionally rich and high-quality dataset. It includes reliable data from 1,852 participants, including standardised 24HDRs, extensive physical measurements, detailed lifestyle questionnaires and rare biosamples, including a biobank for future research. The survey delivers up-to-date information on several nutrition-related health issues (e.g., obesity, hypertension, metabolic disturbances) and provide a unique opportunity to generate laboratory reference values for nutritional biomarkers in a primarily healthy, representative paediatric population. menuCH-Kids thus creates a solid foundation for public health nutrition surveillance, research, and health promotion policy in Switzerland, and can offer valuable information and methodological insights for neighbouring countries. This paper presents the survey methods, data quality, and representativeness of the menuCH-Kids dataset, setting the foundations to future data analyses.

The survey methodological strengths include standardized, multilingual approach covering German-, French- and Italian- speaking regions. It followed European guidelines [19] and used GloboDiet software with an automated multiple-pass method widely used internationally [41–43] ensuring data comparability. Dietary data plausibility is supported by the daily average of 20 foods and 1,934 kcal, which is slightly higher than in the third French survey, INCA3 (18 foods/day and ∼1,726 kcal/day, for 1–17-year-olds) [44]. Under-reporting (15.1%) is in line with INCA3 findings (14%) and Swiss adults (17%) [17], and in the low range of international findings (7%–55% in children with 24HDR [45]). Overweight/obesity prevalence (13%) aligns with prior Swiss findings (15% in 6–12-year-olds, 2023 [14]). In 14–17-year-olds, occasional smoking (11%) and alcohol consumption (36%) closely match the Swiss HBSC results (9.7% and 34.1%, respectively) [46, 47].

Based on a random population sample, menuCH-Kids aims at being representative of the Swiss population aged 6–17 years. Participation rate (11.9%) is in line with previous Swiss surveys (e.g., menuCH adults: 15.1% [17]). menuCH-Kids achieved a 99% full completion and a large sample size (N = 1,852), meeting European guidelines [19] and similar studies [44, 48]. Participation was lower than in France or Belgium (37%–54%) [44, 49, 50]. However, most studies did not include biosamples collection or extensive physical measurements, increasing participants burden and requiring study centre visits for biosamples protocols, which limited the flexibility to offer at-home visits, as done in France or Germany [44, 51], or school-based designs as implemented in Italy [52].

### Limitations

Participation bias is a key concern in population studies [53, 54]. Non-participants were older, non-Swiss, from a small household and an urban environment. The imbalanced age distribution as well as the different age-specific participation rates, though corrected by the weighting strategy in the subsequent outcomes analyses, might have influenced the results of the factors influencing participation. Children from lower socioeconomic neighbourhoods were also underrepresented. Disadvantaged children often have less healthy diets and are at a higher risk of obesity and chronic diseases [13, 55–57]. This bias could lead to an underestimation of unhealthy behaviours. menuCH-Kids survey used the Swiss-SEP [22, 23], an area-based proxy available for both participants and non-participants, integrated into the weighting strategy, to mitigate participation bias [58]. While menuCH-Kids high-quality dataset provides the first representative snapshot of diet and nutrition among children and adolescents in Switzerland, further studies are needed to explore dietary patterns and related health factors of underrepresented groups, such as socioeconomically disadvantaged households, individuals with limited interest in nutrition, and those excluded for logistical reasons (e.g., remote mountain areas, non-speakers of a national language).

### Conclusion

In conclusion, menuCH-Kids provides unique, nationally representative data on diet, health, lifestyle and nutritional status of Swiss children and adolescents. The breadth and quality of the collected data and biosamples position this survey as a benchmark for future research and public health initiatives. Although participation rate and potential biases remain challenges, robust sampling and weighting approaches, including adjustment for socioeconomic position, ensures analytical reliability and supports the development of evidence-based nutrition policies in Switzerland and beyond.
