# Supplementary material for: The First Swiss National Nutrition Survey in Children and Adolescents, menuCH-Kids: Study Design, Participants, and Data Quality
Source: Int J Public Health. 2026 Feb 17;71:1609314. doi: 10.3389/ijph.2026.1609314 (PMC12953171; doi:10.3389/ijph.2026.1609314)
Supplement: Supplementary file 1 [file Supplementaryfile1.docx]

**Supplementary material – International journal of public health
The first Swiss national nutrition survey in children and adolescents, menuCH-Kids: study design, participants, and data quality**


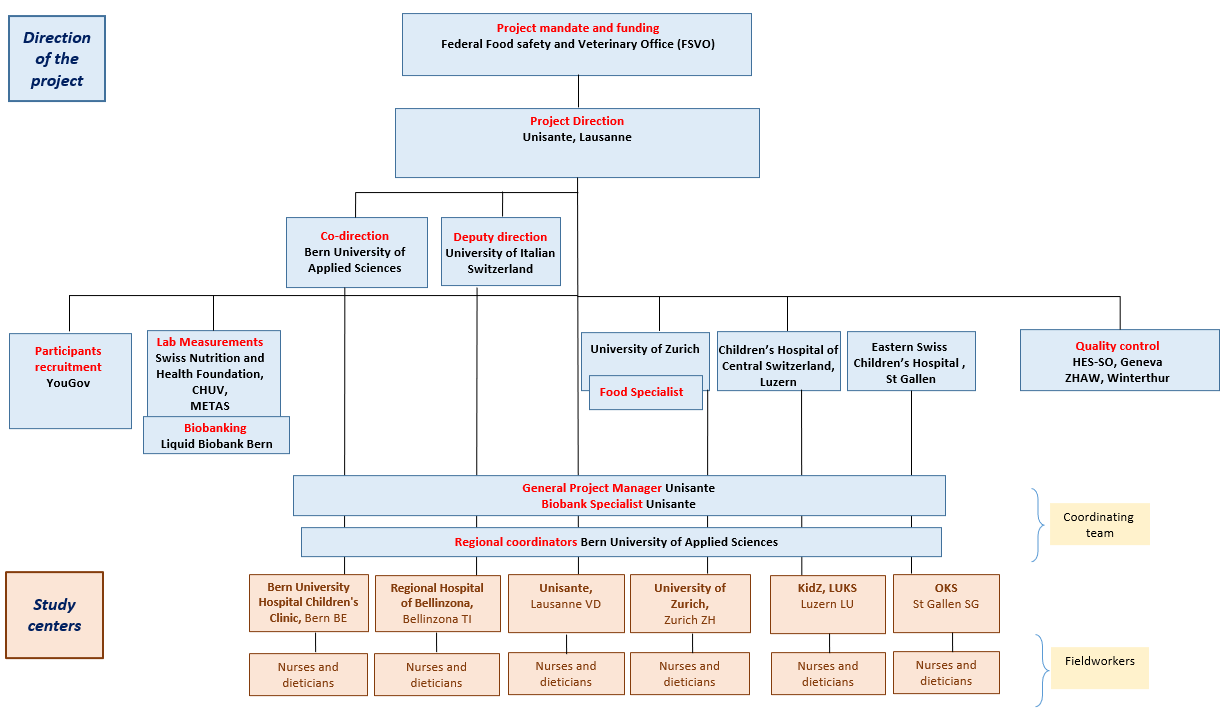


**Supplement 1 |** Organisation chart of the project. menuCH-Kids survey – Switzerland – 2023-2024

**Supplement 2** | Inclusion and exclusion criteria. menuCH-Kids survey – Switzerland – 2023-2024

| **Inclusion criteria** | **Exclusion criteria** |
| --- | --- |
| - Aged 6-17 years |  |
| - Healthy and free-living (not in an institution) | - Known severe chronic disease (diabetes, cancer, Crohn, severe cognitive or developmental disorder, cardiomyopathy …), overnight hospitalization in the last four weeks, pregnancy and breastfeeding |
| - Permanent residents in Switzerland | - Inability to come to study centre |
| - Participant and legal guardian must be able to read and speak the local language (German, French or Italian) | - Insufficient knowledge of language, inability to understand survey |
| - Informed consent signed at the visit | - Inability to provide informed consent, refusal to be informed about incidental findings |

**Supplement 3** | Details about the Swiss Neighbourhood Index of Socioeconomic Position. menuCH-Kids survey – Switzerland – 2023-2024

We used the Swiss Neighbourhood Index of Socioeconomic Position (Swiss-SEP) to estimate the socioeconomic status of both respondents and non-respondents. First developed in 2012, then updated in 2023 with data up to 2015 (1,2), the Swiss-SEP is an area-based index, that assigns a socioeconomic score to each residential building in the country, ranging from 0 (lowest) to 100 (highest) and transformed into quintiles (based on the buildings, not on the population). It is based on neighbourhood-level census and micro-census data, including average rent per square meter, education level, occupation type, and household crowding. These variables were combined using principal component analysis to produce a standardized index. By linking children’s home addresses to the corresponding Swiss-SEP scores, their socioeconomic environment could be approximated even when individual-level data were missing (e.g., for non-respondents), making it a valuable tool for assessing potential bias due to differential response rates.

**Supplement 4** | Online questionnaire. menuCH-Kids survey – Switzerland – 2023-2024

The detailed content of the questionnaires can be found in the documents accompanying the data at *https://www.studydata.blv.admin.ch/catalog/12*. Some questions were taken or inspired from previous surveys, mainly HBSC, menuCH-Kids feasibility study, menuCH adults, BNFCS (Belgium), KIGGS (Germany), aha! Foundation on allergies, FSVO Survey on Food supplements and Promotion Santé Suisse-Gesundheitsförderung Schweiz to facilitate comparisons.

**Supplement 5** | Details about dietary assessments. menuCH-Kids survey – Switzerland – 2023-2024

The picture book and the GloboDiet software were already used in menuCH 2014-2015 (adults), but some updates were done. In the picture book, the content did not change, but pictures order was changed to be grouped by food type. It contains 110 series of four to six portion-size pictures, 19 bread shapes, as well as a thickness chart, a two-dimensional ruler and 12 series of household measures with filling level indication. In GloboDiet, more than 300 new foods were added, mainly children’s specific foods (cookies, sweets…) or new food compared to 2015 (e.g. gluten- and lactose-free products, protein-enriched foods, new sodas, wholemeal products), as well as more than 500 new brand names, more than 600 new commercial food portions and almost 60 new descriptors for foods (e.g. new flavours), based on foods sold in Switzerland as well as dietitian feedback from the interviews.

To calculate precise energy and nutrient intakes, all foods and recipes entered in GloboDiet were matched after the data collection with the closest corresponding generic foods or recipes of the Swiss Food Composition Database (SFCDB), version 6.5.3, using the matching wizard of the FoodCase data management system (3,4). Matching accounted for the name of the food/ingredient/recipe, but also its characteristics (e.g. the descriptors used for preservation, preparation and cooking methods, physical form, sugar or fat content, brand). When importing data into FoodCase, matches from previous matchings were copied, including the pilot data, and the rest were matched semi-automatically. Based on a complex decision tree, all matched foods were then verified by several dietitians and food database specialists. All foods in the SFCDB available for matching with the menuCH-Kids data are also classified according to the food classification and description system FoodEx2 of the European Food Safety Authority EFSA (5).

Finally, all foods were classified according to the updated Swiss Food Pyramid (SFP, 2024) (6). The SFP levels were further divided into sublevels and sub-sublevels according to the nutritional profile of food, enabling their categorisation within the food groups referenced in the pyramid’s dietary recommendations. This procedure was carried out following the same principles applied to the classification of menuCH 2014–2015 data, which was based on the Swiss Food Pyramid in force at that time (2011) (7). The classification was done by 3 experts independently and results were compared, discussed and merged. Details of the classification can be found on *https://www.studydata.blv.admin.ch/catalog/12.*

**Supplement 6 |** Misreporting calculations. menuCH-Kids survey – Switzerland – 2023-2024

Plausibility of 24HDR was assessed by calculating under- and over-reporters’ prevalence, applying Goldberg method adapted by Black (8,9). Energy intake (EI) was taken as the mean of two 24HDRs or as the single value for 12 (0.6%) participants with only one record, with Goldberg’s equation adapted accordingly. Basal Metabolic Rate (BMR) was computed using Schofield’s equations based on weight, height, age group (6-9 ; 10-17), and sex (10). Physical activity level (PAL) was estimated from questionnaire data by the number of days during which they were physically active for at least one hour in the previous week. Participants were classified as low (1-2d/week), medium (3-6d/week) and high (7d/week) PAL, corresponding to values of 1.4-1.6-1.8 for 6-9-year-olds and 1.6-1.8-2.0 for 10-17-year-olds, as recommended by EFSA (11). Medium PAL was assigned for missing and unplausible (e.g “0 day”) PA data (N=51), following EFSA’s PanEU survey recommendation (12).

Additional ways of calculating misreporters with different PAL classifications have been assessed as a quality control and sensitivity analyses. Under-reporters vary between 14 and 18.5% and over-reporters between 0.4% and 1.2%.

In the chosen approach, PAL was estimated by the number of days during which participants were physically active for at least one hour in the last week, with low: 1-2d, medium: 3-6d, high:7d, corresponding to PAL values of 1.4-1.6-1.8 for 6-9-year-olds and 1.6-1.8-2.0 for 10-17-year-olds. Other approaches include:

1. Using the same question about the number of days moving one hour, but with different groupings of days: low= 1-2days/week, medium = 3-4days/week and high = 5-7days/week. Nevertheless, as the recommendation of WHO is 60 minutes of moderate to vigorous physical activity (13), we preferred keeping in “high” only children moving enough every day, even if we did not have information on the intensity of the physical activity.
2. Using another question from the online questionnaire, which sums up the mean time spent moving in six different settings on a regular day: commuting to school, physical education classes, classroom movement, breaks, after-school activities, and chores, and then classifying participants into low, medium and high using the tertiles values. Nevertheless, as data-driven categories can be more biased, we favoured the other question.
3. Putting medium PAL value for all participants (1.6 or 1.8 depending on the age). Nevertheless, the recommendation is to account for physical activity.

| **PAL calculation** | **Under-reporters** | **Over-reporters** |
| --- | --- | --- |
| **Chosen method** | **15.1%** | **0.6%** |
| Option a) | 18.5% | 0.4% |
| Option b) | 15.7% | 1.2% |
| Option c) | 14.0% | 0.6% |

**Supplement 7** | Clothing weights. menuCH-Kids survey – Switzerland – 2023-2024

Several clothing types (pants, t-shirts, sweaters, shorts, skirts, leggings, joggings, dresses, underwear) were weighted in six different “age-sizes” and then several complete outfits were created and used to estimate a mean clothing weight in the four clothing-categories below, for each age range. They were then subtracted from the measured weight.

|  | **Weight clothes**  **6-9-years-olds**  **in grams (g)** | **Weight clothes**  **10-13-years-olds**  **in grams (g)** | **Weight clothes**  **14-17years-olds**  **in grams (g)** |
| --- | --- | --- | --- |
| Underwear = code 1 | 80 | 80 | 80 |
| Light clothes = code 2 | 300 | 360 | 410 |
| Jeans and t-shirt = code 3 | 400 | 630 | 740 |
| Heavy clothes = code 4 | 500 | 820 | 930 |

**Supplement 8** **|** Overview of blood and urine collection and analyses. menuCH-Kids survey – Switzerland – 2023-2024

Spot urine was collected at home using a cup sent by post with hygiene instructions. First morning urine and storage in the refrigerator were recommended. For first morning urine, a cutoff time for collection was set at noon, unless otherwise justified in a comment. If a night void occurred before 5 a.m. and more than 2 hours before waking up, it still counted as a first morning urine. At the centre, ~17 ml of urine was aliquoted into nine tubes and frozen at –80°C, with temporary -20°C storage if needed (e.g. during weekend).

Voluntary blood sampling was performed by a paediatric nurse, with 21.8 ml of venous blood collected in six tubes (2 serum, 3 EDTA and 1 Lithium-Heparin). Tubes were placed in a closed cooler box immediately, except for serum tubes kept at room temperature for coagulation. Two of the EDTA tubes were analysed directly on-site for blood formula and glycated haemoglobin (HbA1c). Four tubes for biobanking were processed in 26 aliquots, frozen at -80°C within ~1hour. Five overnight frozen shipments from the centres to the central biobank (LBB) were made during the main phase, followed by batch analyses of some aliquots and long-term storage of others (=biobanking). Several batch analyses for nutritional status and contaminants have been done in blood (vitamins A, E, B1, B2, B6, C, lipids, ferritin, calcium, selenium, zinc, copper, lead, cadmium, arsenic, copper, PFAS) and in urine (iodine, bisphenols, phthalates).

*
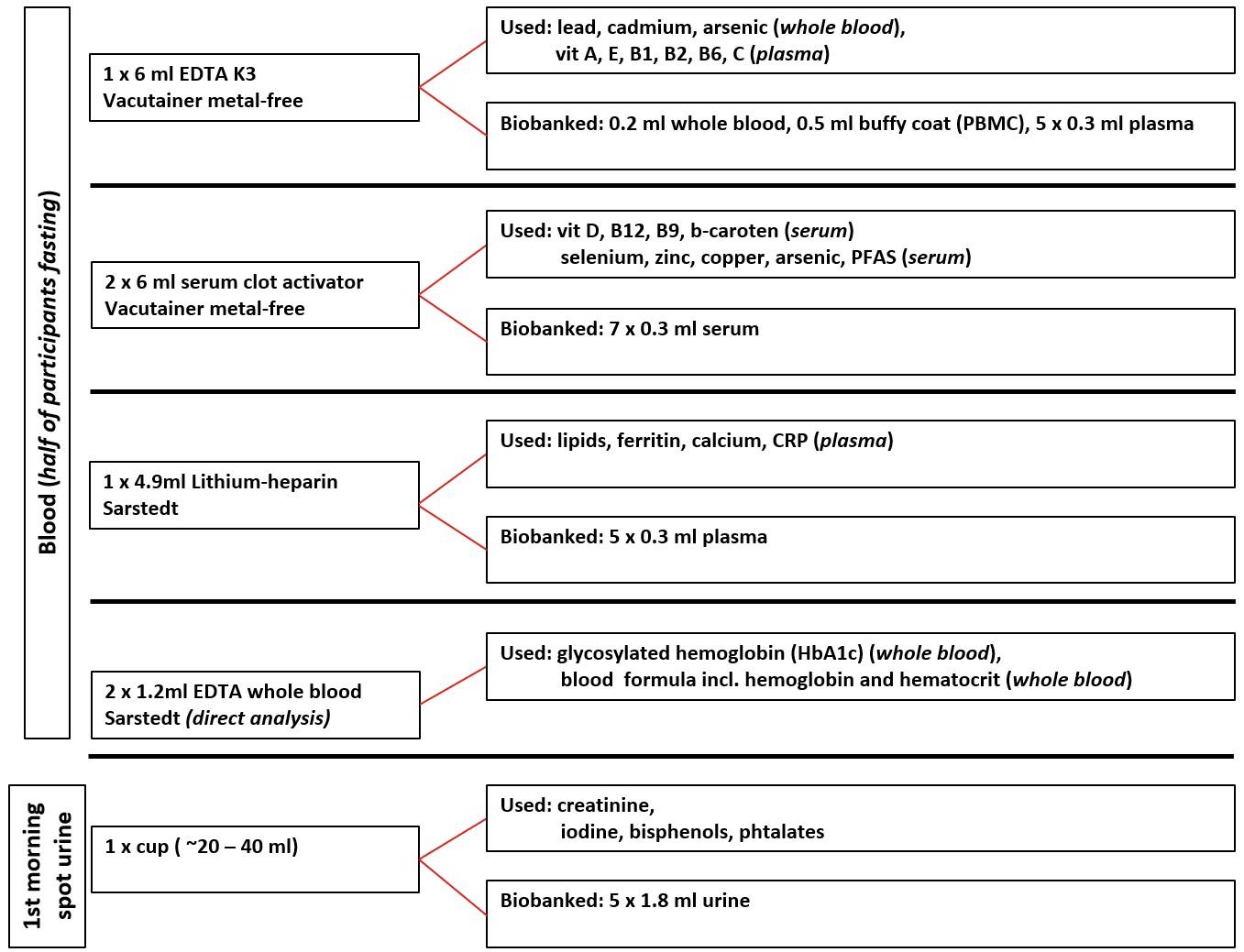
*

**Supplement 9** | Detailed weighting strategy. menuCH-Kids survey – Switzerland – 2023-2024

Three sets of weights were developed to make the participant sample comparable to the Swiss population. The first set was calculated for participants who attended the on-site visit (N = 1,852 participants), the second set was for those who consented to blood sample collection (N = 848 participants) and the third for all participants with a questionnaire, including those without an on-site visit (N = 1,935). The weight calculation process was similar for all sets of weights and can be summarized in four steps.

In the first step, sampling weights were calculated to reflect the sampling methodology. The sampling was conducted in four waves across 18 strata (six centres by three age groups), with population totals available for each stratum at each wave. To simplify the weight calculation, data from the four waves were pooled and an average sampling frame was calculated by averaging the population totals over the four waves. The sampling weights were defined as the inverse of the inclusion probabilities, which were assumed equal for all individuals within the same stratum. The inclusion probabilities were defined as the ratio of the total number of individuals invited to participate in a given stratum to the size of the average sampling frame for that stratum.

The second step involved correcting the sampling weights obtained at step one for non-response. This adjustment was achieved by modelling the response probability based on variables that were used to define the strata (i.e. age group and study centre) as well as sex, nationality, household size, and Swiss-SEP quintiles using a logistic regression model. Initially, all predictors and their pairwise interactions were included in the model. A backwards elimination process based on the Bayesian Information Criterion (BIC) (14) was then applied to refine the model, retaining only those predictors and interactions whose removal would result in a higher BIC (indicating a poorer fit). The predicted response probabilities from the final model were then analysed using a hierarchical clustering algorithm (k-means) to build up to five homogeneous response groups. The common response probability in each group was calculated as the weighted average of the participation indicator (1 for participants, 0 for non-participants) for all individuals in the group, using the sampling weights obtained at the first step. The weights corrected for non-response were then computed as the ratio of the sampling weights obtained in step one to the common response probability for each individual’s group (defined by k-means).

In the third step, the weights obtained after non-response correction were used to calibrate the sample to align with population totals for design and auxiliary variables through a raking procedure executed in two phases (15). First, a preliminary calibration was performed to align the sample with population totals for the 7 major regions of Switzerland. The resulting weights were then adjusted for the remaining variables: sampling strata (18 categories which combine age groups and study centres), age (separate years), sex, nationality, household size, Swiss SEP quintiles, and season. The process was then repeated until convergence. This sequential calibration approach was adopted because the raking algorithm often had difficulty converging when both the 18 strata and the 7 major regions of Switzerland were included simultaneously, as the strata information (which includes the area around the study centre) is largely colinear with the regional information. This method ensured that the strata margins were matched while still achieving adequate calibration for the regions. Note that the four seasons were defined based on the month of the visit with winter covering the months December to February. The margins for each season were calculated by dividing the size of the average sampling frame by four. To account for difference in dietary intake during the week, the day of the week was used as an additional auxiliary variable in the calibration process for the first set of weights only (participants who attended the on-site visit). The day of the week was categorized into three groups (week=Monday to Thursday, weekend=Friday to Sunday, mix of both) based on the days of the first and second 24HDRs. Participants with a single 24HDR were allocated to the “week” or “weekend” categories based on the day of their unique 24HDR. The margins for the day of the week were obtained by multiplying the size of the average sampling frame by 16/49 (week), 9/49 (weekend) or 24/49 (mix), corresponding to the combinations of ordered pairs of days of the two weeks falling into each category.

Finally, the last step involved trimming excessively large weights that could adversely affect the variance of the weighted estimator. A trimming limit was set at the median plus five times the interquartile range of the weights. Any weight exceeding this limit was capped at the limit, and the remaining weights were adjusted proportionally to ensure the sum of the weights still equals the size of the sampling frame. When weight trimming was necessary, the calibration process (step three) was repeated on the trimmed weights to ensure proper calibration. This process of weight trimming and calibration was carried out sequentially until no weight exceeded the trimming limit.

A rescaling bootstrap procedure based on the approach proposed by Rao and Wu (1988) was used to build 1000 replicates for each set of weights (16). These replicates were used to calculate the variance of the weighted estimators while accounting for the sampling design.

To use the provided weights, all participants need to be included, even those having missing data in some variables, as excluding participants based on missingness would require specific weights each time. Missing data is low: <1% for all anthropometry variables, except for blood pressure for which 1.9% of participants don’t have the three measures (but only 0.5% have none) ; <1.2% for all questionnaire variables (when accounting for branching logics) ; and for blood, direct analyses have up to 5.8% missing, but most batch analyses have ~3.5% missing, and vitamins have only 1.6% of missing values.

To impute those values, we suggest using multiple imputation by chained equations (MICE) (17), for its flexibility with variable types and ability to model complex interdependencies. Ten datasets can be generated, and final estimates pooled using Rubin’s rules to account for imputation and sampling uncertainty. A possible choice for the imputation model includes 56 variables capturing relevant socio-demographic and health information:

| **Variable name** | **Variable description** |  |
| --- | --- | --- |
| fso_yearofbirth | Year of birth |  |
| age_pkg | Age |  |
| fso_householdsizesrph | Household size |  |
| fso_sex | Sex |  |
| fso_maritalstatus | Participant’s marital status |  |
| fso_nationalitystate | Participant’s nationality |  |
| fso_residenceswisszipcode | Swiss zip code of residence |  |
| fso_communicationlanguage | Participant’s spoken language |  |
| fso_communelanguage | Official language of the commune lived in |  |
| fso_strate | Participant’s age and centre group |  |
| fso_package_no | Participation package number |  |
| fso_typo | Residential area |  |
| fso_sep_q | Quantile socioeconomic score |  |
| gd_day_int1 | Weekday on which the visit took place |  |
| mk_f2f_weight_final | Weight after clothes subtraction |  |
| mk_f2f_height | Measured height |  |
| mean mk_f2f_abdominal_circ_1 / 2 /3 | Waist circumference (mean 3 measures) |  |
| mean mk_f2f_hip_circ_1 1/2/3 | Hip circumference (mean 3 measures) |  |
| mk_f2f_skin_scan_skintyp | Skin type |  |
| mk_f2f_puberty_boy_gd *OR*  mk_f2f_girl_puberty_bd | Genital development stage (for boys) or  participant’s breast development stage (for girls) |  |
| mk_f2f_fish_7d___1 | Consumption of fish salmon type |  |
| mk_f2f_fish_7d___2 | Consumption of other type of fish / white fish |  |
| mk_f2f_fish_7d___3 | Consumption of sea food |  |
| mk_f2f_fish_7d___4 | No fish consumption / doesn’t remember |  |
| mck_f2f_outside_week | Average time spent outside/day (week) |  |
| mck_f2f_outside_weekend | Average time spent outside/day (weekend) |  |
| mean mk_f2f_sbp_1 /2 /3* | Mean measured systolic blood pressure |  |
| mean mk_f2f_dbp_1 /2/3* | Mean measured diastolic blood pressure |  |
| mean mk_f2f_heart_rate_1 / 2 / 3 | Mean measures heart rate |  |
| mk_f2f_fasting | Fasting status |  |
| hba1cs | Hba1c value (calibration ngsp-dcct) (%) |  |
| hba1ci | Hba1c value (calibration ifcc) (nmol / mol) |  |
| hb | Haemoglobin value (g / l) |  |
| ht | Haematocrit value (%) |  |
| mch | Mean corpuscular content in haemetocrit (pg) |  |
| mchc | Mean corpuscular concentration in haematocrit (g/l) |  |
| mcv | Average globular volume (fl) |  |
| plaq | Blood platelets (g / l) |  |
| rdw | Red cell distribution width (%) |  |
| ery | Erythrocytes (T / l) |  |
| leuc | Leucocytes (G / l) |  |
| pdw | Index of platelets distribution (fl) |  |
| mpv | Average platelets volume (fl) |  |
| nrbc | Erythroblasts / 100 leucocytes |  |
| chol | Total cholesterol value (mmol / l) |  |
| chdl | Hdl cholesterol value (mmol / l) |  |
| ldl | Ldl cholesterol value (mmol / l) |  |
| tg | Triglycerides value (mmol / l) |  |
| crp | Crp value (mg / l) |  |
| frt | Ferritin value (ug / l) |  |
| ca | Calcium value (mmol / l) |  |
| vitd | Vitamin d value (nmol / l) |  |
| vitb9 | Plasma vitamin b9 value (nmol / l) |  |
| vitb12 | Vitamin b12 value (pmol / l) |  |
| bcar | Β-carotene value (nmol / l) |  |
| urct | Urine creatinine value (µmol/l) |  |
| ** The mean of the two last measures was used (if only two measures were taken, the mean was used, unless the difference was bigger than 20 mmHg for systolic / 10mmHg for diastolic, in which case only the second measure was taken).* | | |

**Supplement 10 |** Odds ratios and 95% confidence intervals for factors associated with participation**.** menuCH-Kids survey – Switzerland – 2023-2024

| **Term** | **M1** | **M2** |
| --- | --- | --- |
| Age 6–9 (ref) |  |  |
| Age 10–13 | 1.14 (1.01–1.28)* | 1.14 (1.01–1.28)* |
| Age 14–17 | 0.83 (0.74–0.93)** | 0.83 (0.74–0.93)** |
| Vaud (ref) |  |  |
| Bern | 1.07 (0.92–1.24) | 1.06 (0.91–1.23) |
| Lucerne | 1.06 (0.89–1.26) | 1.08 (0.90–1.27) |
| St. Gallen | 1.10 (0.94–1.30) | 1.15 (0.98–1.36) |
| Ticino | 1.05 (0.88–1.25) | 1.20 (1.00–1.43) |
| Zurich | 1.00 (0.85–1.17) | 0.89 (0.76–1.05) |
| Female (ref) |  |  |
| Male | 0.90 (0.82–0.99)* | 0.90 (0.82–0.99)* |
| Swiss (ref) |  |  |
| Other nationality | 0.45 (0.39–0.51)*** | 0.48 (0.42–0.55)*** |
| Household size: 2 | 0.48 (0.34–0.65)*** | 0.50 (0.36–0.68)*** |
| Household size: 3 | 0.79 (0.68–0.91)** | 0.79 (0.68–0.91)** |
| Household size: 4 (ref) |  |  |
| Household size: 5 | 1.10 (0.98–1.24) | 1.11 (0.99–1.26) |
| Household size: 6+ | 0.90 (0.75–1.08) | 0.93 (0.78–1.11) |
| Urban (ref) |  |  |
| Suburban | 1.05 (0.93–1.18) | 1.09 (0.97–1.23) |
| Rural | 1.20 (1.03–1.39)* | 1.38 (1.18–1.60)*** |
| Swiss-SEP Q1 | - | 0.48 (0.41–0.58)*** |
| Swiss-SEP Q2 | - | 0.65 (0.55–0.77)*** |
| Swiss-SEP Q3 | - | 0.78 (0.67–0.92)** |
| Swiss-SEP Q4 | - | 0.82 (0.70–0.95)** |
| Swiss-SEP Q5 (ref) | - |  |

** p < 0.05, ** p < 0.01, *** p < 0.001.* M1: basic model predicting participation including age, centre, sex, nationality, household size and residential area; M2: Model adding Swiss-SEP *Reference categories: age group 6–9 years, female sex, Vaud centre, Swiss nationality, household size of 4, urban residence, and highest Swiss-SEP quintile (Q5).*

**References**

1. Panczak R, Galobardes B, Voorpostel M, Spoerri A, Zwahlen M, Egger M. A Swiss neighbourhood index of socioeconomic position: development and association with mortality. J Epidemiol Community Health. 2012 Dec;66(12):1129–36.

2. Panczak R, Berlin C, Voorpostel M, Zwahlen M, Egger M. The Swiss neighbourhood index of socioeconomic position: update and re-validation. Swiss Medical Weekly. 2023 Jan 12;153(1):40028–40028.

3. Federal Food Safety and Veterinary Office. The Swiss Food Composition Database. [cited 2022 Aug 12]. Swiss Food Composition Database. Available from: https://valorinutritivi.ch/en/

4. Presser K, Weber D, Norrie M. FoodCASE: A system to manage food composition, consumption and TDS data. Food Chemistry. 2018 Jan 1;238:166–72.

5. Authority (EFSA) EFS. The food classification and description system FoodEx 2 (revision 2). EFSA Supporting Publications. 2015;12(5):804E.

6. FSVO. Swiss Dietary Recommendations [Internet]. [cited 2025 Sept 8]. Available from: https://www.blv.admin.ch/blv/en/home/lebensmittel-und-ernaehrung/ernaehrung/empfehlungen-informationen/schweizer-ernaehrungsempfehlungen.html

7. Chatelan A, Beer-Borst S, Randriamiharisoa A, Pasquier J, Blanco JM, Siegenthaler S, et al. Major Differences in Diet across Three Linguistic Regions of Switzerland: Results from the First National Nutrition Survey menuCH. Nutrients. 2017 Oct 25;9(11):1163.

8. Goldberg GR, Black AE, Jebb SA, Cole TJ, Murgatroyd PR, Coward WA, et al. Critical evaluation of energy intake data using fundamental principles of energy physiology: 1. Derivation of cut-off limits to identify under-recording. Eur J Clin Nutr. 1991 Dec;45(12):569–81.

9. Black AE. Critical evaluation of energy intake using the Goldberg cut-off for energy intake:basal metabolic rate. A practical guide to its calculation, use and limitations. Int J Obes Relat Metab Disord. 2000 Sept;24(9):1119–30.

10. Schofield WN. Predicting basal metabolic rate, new standards and review of previous work. Hum Nutr Clin Nutr. 1985;39 Suppl 1:5–41.

11. EFSA Panel on Dietetic Products, Nutrition and Allergies (NDA). Scientific Opinion on Dietary Reference Values for energy. EFSA Journal. 2013;11(1):3005 (Appendix 13).

12. Ambrus Á, Horváth Z, Farkas Z, Dorogházi E, Cseh J, Petrova S, et al. Pilot study in the view of a Pan-European dietary survey – adolescents, adults and elderly. EFSA Supporting Publications. 2013;10(11):508E.

13. WHO. guidelines on physical activity and sedentary behaviour [Internet]. 2020 [cited 2025 Sept 9]. Available from: https://www.who.int/publications/i/item/9789240015128

14. Schwarz G. Estimating the Dimension of a Model. Ann Statist [Internet]. 1978 Mar 1 [cited 2025 Apr 29];6(2). Available from: https://projecteuclid.org/journals/annals-of-statistics/volume-6/issue-2/Estimating-the-Dimension-of-a-Model/10.1214/aos/1176344136.full

15. Deville JC, Särndal CE. Calibration Estimators in Survey Sampling. Journal of the American Statistical Association. 1992 June;87(418):376–82.

16. Rao JNK, Wu CFJ. Resampling Inference with Complex Survey Data. Journal of the American Statistical Association. 1988 Mar;83(401):231–41.

17. White IR, Royston P, Wood AM. Multiple imputation using chained equations: Issues and guidance for practice. Statistics in Medicine. 2011;30(4):377–99.
